# Effects of High Hydrostatic Pressure Processing on Potential Allergenicity and Quality of Tropomyosin in Pre-Prepared Crab Products (*Portunus trituberculatus*)

**DOI:** 10.3390/foods15193472

**Published:** 2026-09-28

**Authors:** Surong Huang, Min Xu, Tingru Chen, Shuilin Cai, Zhiyu Liu

**Affiliations:** 1 College of Food Science, Fujian Agriculture and Forestry University, Fuzhou 350002, China; huang_sr_77@163.com; 2 Key Laboratory of Cultivation and High-Value Utilization of Marine Organisms in Fujian Province, National Research and Development Center for Marine Fish Processing, Fisheries Research Institute of Fujian, Xiamen 361013, China; xumin1315@foxmail.com (M.X.); ctr1003533034@163.com (T.C.); caishuilin@gmail.com (S.C.)

**Keywords:** swimming crab cake, high hydrostatic pressure, immunoreactivity, quality

## Abstract

This study investigated the effects of high hydrostatic pressure (HHP; 200 MPa for 5–15 min) on tropomyosin (TM) immunoreactivity, in vitro digestive stability, and selected quality attributes of crab cakes prepared from *Portunus trituberculatus*. Western blotting and competitive indirect ELISA (ic ELISA) revealed that 200 MPa treatment significantly reduced TM IgG binding capacity, with the greatest reduction (21.19% decrease, *p* < 0.05) at 10 min. In vitro digestion showed that TM degraded most rapidly in simulated gastric and intestinal fluids following 200 MPa for 10 min. HHP treatment significantly enhanced water-holding capacity by converting free water to bound water, with optimal retention at 200 MPa for 10 min. Texture analysis indicated that the treatment at 200 MPa for 10 min showed outstanding performance in chewiness and made the crab cakes brighter and more reddish-yellow in color. Sensory evaluation confirmed that HHP-treated samples closely resembled conventionally cooked products in flavor and taste, exhibiting good acceptability. Overall, 200 MPa for 10 min was identified as the optimal HHP treatment condition under the present experimental conditions, as it significantly reduced the IgG-binding capacity of TM while synergistically improving water-holding capacity, texture, color, flavor, and taste stability of crab cakes. Therefore, this provides a feasible technical pathway for the development of high-quality pre-prepared crustacean foods with reduced immunoreactivity.

## 1. Introduction

*Portunus trituberculatus* (swimming crab), belonging to the family Portunidae, is one of China’s most important economic marine species. Its tender, flavorful flesh and high nutritional profile make this species popular among consumers [1,2]. According to the 2024 statistical data, the total mariculture production of swimming crab in China reached 97,000 tons, of which Fujian Province alone accounted for 34,000 tons, representing approximately 35% of the national total [3]. Despite its economic significance, the industry faces supply chain constraints due to spatial and seasonal limitations, while growing consumer demand for convenient, safe, and high-quality ready-to-eat seafood drives the need for advanced processing technologies. However, conventional thermal sterilization, while effective at microbial control, often adversely affects crab meat texture, characteristic flavor, and nutritional constituents, thereby limiting the quality and market competitiveness of pre-prepared crab products [4].

The swimming crab is a common food-borne allergen, with its allergic reactions primarily mediated by immunoglobulin E (IgE) and classified as a type I immediate hypersensitivity reaction [5]. Clinical manifestations of this allergy can range from mild urticaria and allergic rhinitis to life-threatening anaphylactic shock, often persisting lifelong [6]. Tropomyosin (TM) is regarded as the principal allergen across all edible crustacean products [7]. TM is highly conserved among crustacean species, providing a molecular basis for interspecies cross-reactivity [8,9]. Critically, TM exhibits pronounced heat stability [10,11]; conventional cooking processes are insufficient to completely inactivate it, allowing it to retain IgE-binding capacity and trigger immune responses [12]. Consequently, residual TM immunoreactivity represents an important consideration in the safety assessment of precast crustacean products. Therefore, the development of novel processing technologies that can both improve product quality and control allergens has become a critical breakthrough for the industrial upgrading of pre-prepared crab products.

High Hydrostatic Pressure (HHP), as an emerging non-thermal processing technology, utilizes water or other liquids as the pressure-transmitting medium to impose a static pressure of 100–1000 MPa on food matrices within a sealed vessel for a specified duration [13]. This pressure is applied uniformly and instantaneously, generating minimal heat and thereby preserving the intrinsic quality of the food [14,15]. HHP can also induce structural modifications in proteins and muscle tissues. This technology can improve product texture and color while causing minimal damage to heat-sensitive small molecules, thus retaining product flavor to a greater extent.

Notably, HHP technology has also shown great potential in controlling food allergens, as it reduces allergenicity by altering the spatial conformation of allergenic proteins rather than degrading their primary structure—a unique mechanism that confers a distinct advantage over traditional thermal processing in allergen control. Research by Zhou et al. demonstrated that pressures exceeding 250 MPa induced significant structural alterations in the myofibrillar proteins of *Scylla paramamosain,* leading to increased surface hydrophobicity and consequently reduced allergenicity [16]. Liu et al. also compared the effects of steaming, boiling, and HHP on *Scylla serrata* muscle tissue, finding that HHP effectively reduced the allergenicity of crab muscle crude protein by decreasing the content and diversity of allergens and disrupting the protein’s spatial structure [17]. HHP treatment can also fundamentally attenuate allergenic potential by disrupting the spatial conformation of TM, exposing previously buried proteolytic cleavage sites and accelerating its hydrolysis in the digestive tract [18]. Further investigations by Jin et al. indicated that HHP-treated TM was degraded more rapidly and thoroughly during simulated gastrointestinal digestion, with a significant reduction in intact protein content. This diminished digestive stability directly confirmed its reduced allergenicity [19]. Notably, *Scylla paramamosain* contains other potential allergens, including sarcoplasmic calcium-binding protein (SCP) [20], myosin light chain (MLC), and arginine kinase (AK) [7]. Previous studies have also shown that combined thermal and HHP treatment can mask the linear epitopes of AK, hindering its IgE binding and reducing allergenicity [21,22], suggesting broad applicability of this technology in allergen control. Beyond reducing allergenicity, HHP treatment profoundly impacts product sensory attributes and quality [23]. Studies indicate that HHP can effectively modulate the color, texture characteristics, and flavor profile of meat products by altering the supramolecular structure of muscle proteins, regulating lipid oxidation status, and remodeling the microstructure of muscle fibers [24]. For instance, in crustacean seafood, 200–300 MPa HHP treatment promotes partial depolymerization of the collagen network in connective tissues, thereby enhancing meat tenderness and juiciness [18,25]. Although HHP itself does not directly induce the Maillard reaction, when used synergistically with mild thermal treatment, it can indirectly enhance the generation of characteristic flavor compounds by releasing more free amino acids and reducing sugars and promoting the restructuring of lipid oxidation products [26].

Although the above studies indicate that HHP holds great promise in allergen control, most existing research has been conducted using purified proteins or simple buffer systems, which fail to fully account for the complexity of real food matrices. Notably, during the actual processing of pre-prepared crab products, the complex food matrix—composed of lipids, polysaccharides, and ionic constituents—may interfere with the conformation and epitope exposure of TM through physical masking, chemical modification, or intermolecular interactions, leading to discrepancies between the desensitization patterns observed in purified systems and those in actual complex food matrices. Our preliminary experiments observed that HHP treatment at 200 MPa significantly reduced the immunoglobulin-binding capacity of TM in *Portunus trituberculatus*. However, whether this effect holds true in the crab cake system, and how treatment duration influences the immunoreactivity and overall quality of the final product, remains unclear. To address these knowledge gaps, this study employed crab cakes prepared from *Portunus trituberculatus* as the research model. HHP treatment at 200 MPa was applied for different durations (5, 10, and 15 min). The immunoreactivity of tropomyosin in crab cakes was systematically evaluated using Western blot, indirect ELISA, and indirect dot blot following simulated gastrointestinal digestion. Meanwhile, water-holding capacity, moisture migration, textural properties, color, and flavor profiles were comprehensively analyzed. The results provide a comprehensive understanding of the effects of HHP pretreatment on the potential allergenicity and overall quality of crab cakes. This study aims to establish a theoretical foundation for the development of high-quality and safer crab meat products.

## 2. Materials and Methods

### 2.1. Materials and Reagents

*Portunus trituberculatus* meat was obtained from Hongxin Aquatic Products Flagship Store; transglutaminase (TGase) was purchased from Zhongxing Biotechnology; hydroxypropyl di-starch phosphate (HDP) was sourced from Zhongyuan Biotechnology; dithiothreitol (DTT) was obtained from Beijing Vander Biotechnology Co., Ltd. (Beijing, China); sodium dodecyl sulfate (SDS) and tris(hydroxymethyl)aminomethane (Tris) were purchased from Shanghai Aladdin Biochemical Technology Co., Ltd. (Shanghai, China); 5× protein loading buffer, single-component TMB chromogenic solution, and ELISA stop solution were procured from Beijing Solarbio Science & Technology Co., Ltd. (Beijing, China); molecular weight marker, Tween-20, and antibody diluent were obtained from Beijing LABLEAD Inc. (Beijing, China); Ocean University of China supplied rabbit polyclonal anti-TM antibody; HRP-conjugated goat anti-rabbit IgG antibody was purchased from Xiamen Taijing Biotechnology Co., Ltd. (Xiamen, China).

### 2.2. Crab Cake Processing Technology

Crab meat (60%) and vegetable puree (40%) were used as the primary ingredients for crab cakes. Subsequently, auxiliary ingredients were incorporated based on the weight of the primary ingredients: HPD (9%), salted egg yolk (3%), minced ginger (3%), black vinegar (2%), white sugar (1.5%), TGase (0.2%), and salt (0.1%). These ingredients were mixed thoroughly in a basin. The homogenized crab mixture was then placed in a container and refrigerated at 4 °C for 1 h. The rested crab mixture was then molded into patties and baked in an oven at 180 °C (top and bottom heat) for 10 min. Following baking and cooling, the crab cakes were vacuum-packaged and subjected to HHP at 200 MPa for 5, 10, and 15 min, designated as the 200-5, 200-10, and 200-15 groups, respectively. A group of HHP-untreated crab cakes was maintained as a control, designated as the CK group. Each experimental group comprised at least three replicates.

### 2.3. Preparation of Crude Protein from TM Crab Cakes

The method of Sun et al. was slightly modified [27]. In short, 10 g of crab cake was weighed and combined with 5 volumes (*w*/*v*) of pre-chilled Buffer B (containing 0.02 mol/L Tris-HCl, 1 mol/L KCl, and 0.1 mmol/L DTT). The mixture was homogenized in an ice-water bath for 3 min, followed by incubation with shaking at 4 °C for 24 h to ensure complete extraction. Following extraction, the homogenate was centrifuged at 7000× *g* for 30 min at 4 °C. Finally, the supernatant was carefully collected to obtain the crude protein extract solution from the crab cakes.

### 2.4. SDS-PAGE and Western Blot Analysis

The total protein concentration of each sample was determined by the Bradford method, and all samples were diluted to the same concentration prior to electrophoresis to ensure equal loading. SDS-PAGE was performed using a 12% resolving gel and a 5% stacking gel, and electrophoresis was conducted at a constant voltage of 160 V until the bromophenol blue front reached the bottom of the gel. After electrophoresis, the gel was stained with Coomassie Brilliant Blue R-250 solution for 10 min at room temperature, followed by destaining with deionized water. The gel was then scanned using a protein gel scanner for imaging.

For Western blot analysis, the gel strip corresponding to approximately 40 KDa (the theoretical molecular weight of TM) was excised based on the migration position of the pre-stained protein molecular weight standards, and then transferred to a PVDF membrane. Protein transfer was performed at 100 V for 90 min under ice-cold conditions. After the transfer, the membrane was incubated in PBST buffer containing 5% non-fat dry milk at room temperature for 2 h for blocking. Primary antibody (rabbit anti-TM polyclonal antibody, 1:20,000 dilution) was added and incubated overnight at 4 °C, followed by incubation with secondary antibody (HRP-conjugated goat anti-rabbit antibody, 1:20,000 dilution) at room temperature for 2 h. After washing with PBST, the membrane was visualized using a chemiluminescent substrate (ECL Super). Band intensity analysis of the target protein was performed using ImageJ software (v1.53a).

### 2.5. Competitive Inhibition ELISA Analysis

Following a modified protocol based on Cheng et al. [28], 100 μL of rabbit anti-TM polyclonal antibody (1:20,000 dilution) was mixed with an equal volume of various samples and incubated at 37 °C for 1 h. The mixture was then added to a 96-well plate pre-coated with TM, incubated at 37 °C for 1 h, and washed with PBST. Subsequently, 100 μL of HRP-conjugated goat anti-rabbit antibody (1:20,000 dilution) was added and incubated at 37 °C for 1 h. Finally, 100 μL of TMB one-component chromogenic substrate was added to each well and incubated in the dark at 37 °C for 15 min, followed by the addition of 50 μL of stop solution to terminate the reaction. Absorbance values at 450 nm were measured within 30 min, and the competitive inhibition rate (Z) was calculated using In Equation (1).

(1) $$Z\left(\%\right)=\left(1-\frac{X}{Y}\right)\times 100\%$$

In Equation (1), X denotes the absorbance of the sample well; Y denotes the absorbance of the blank well.

### 2.6. In Vitro Gastrointestinal Simulated Digestion of Crab Cakes

The preparation of simulated gastrointestinal fluids followed the method described in the United States Pharmacopeia [29]. Briefly, crab meat was mixed with an equivalent volume of simulated gastric fluid (SGF), achieving a final enzyme activity of 2000 U/mL (pepsin-specific activity of 2354 U/mg protein). The pH was adjusted to 2.0, and the mixture was incubated in a water bath at 37 °C with shaking. Samples were collected at 0, 5, 15, 30, 60, and 120 min. Digestion was terminated by rapidly increasing the pH to 7.4 with NaOH, with the 0 min sample involving pre-inactivated pepsin. The product of 120 min gastric digestion was then mixed with pre-warmed simulated intestinal fluid (SIF) containing trypsin to achieve a final enzyme activity of 2000 U/mL (trypsin-specific activity of 200 U/mg protein) and incubated further at 37 °C. Samples were collected at 0, 5, 15, 30, 60, and 120 min post-digestion initiation. Reactions were terminated by immediate immersion in a boiling water bath for 10 min to inactivate the enzymes, with the 0 min sample involving pre-inactivated trypsin. The control (Con) group, serving as a control, did not include protease, while all other steps remained identical.

#### Dot Blot Analysis of Simulated Digestates

The method described by Lu et al. was slightly modified [14]. In short, a 1 cm × 1 cm NC membrane was moistened with PBS, and TM protein was added dropwise; the membrane was then incubated at 37 °C for 30 min. After washing with PBST, the membrane was soaked in a solution containing 5% skim milk powder and blocked at 37 °C for 2 h. Simulated gastrointestinal digestion products from different time points were mixed with an equal volume of primary antibody, incubated at 37 °C for 1 h, and then washed with PBST. A secondary antibody was added, and incubation continued for 1 h, followed by washing with PBST. Finally, an ECL chemical developing solution was added to the NC membrane for 1 min, and the membrane was developed in an imager.

### 2.7. Water-Holding Capacity

We initially ground the baked and cooled crab cakes using a homogenizer. We weighed 2.00 g of crab cake, denoted as M1 (to the nearest 0.01 g), and placed it on top of a double layer of filter paper lining the bottom of a centrifuge tube. After centrifuging at 7000× *g* for 15 min, we gently blotted any residual free water from the sample surface with filter paper, and then weighed the sample and recorded the weight as M_2_. Then, we calculated the water-holding capacity (WHC) according toIn Equation (2).

(2) $$\mathrm{WHC}\left(\%\right)=\frac{M_{2}}{M_{1}}\times 100\%$$

In Equation (2), M_1_ is the mass of the sample before centrifugation, and M_2_ is the mass of the sample after centrifugation and blotting off surface water.

### 2.8. Moisture Migration

Measurements were performed using low-field NMR following a slightly modified method described by Wang et al. [30], with four parallel measurements taken for each sample. The crab cakes were placed in the instrument’s NMR probe to begin the test. Calibration was performed using an oil sample with an FID sequence, and the moisture distribution was determined using the Carr–Purcell–Meiom–Gill (CPMG) sequence. The specific parameters are as follows: TW = 2500 ms, SF = 21 MHz, SW = 200 KHz, NS = 16, RFD = 0.02 ms, DRG1 = 3, and RG1 = 10 db.

### 2.9. Texture Profile Analysis

Using the full-texture method, the cooked crab cakes were cooled to room temperature and placed on a platform [31]. Moreover, texture profiling was performed using the “TPA” mode of the TA.XT Express. A spherical P/1sp probe was used to conduct two cyclic compression tests on the samples. The pre-test rate was 2 mm/s, the test rate was 2 mm/s, and the post-test rate was 5 mm/s, with a 5-s interval between the two compression cycles and a deformation of 50%. The trigger force for each sample was 5 g. Ten tests were conducted for each group of samples, and the average value was calculated.

### 2.10. Color Measurement

We followed the method described by Wu et al., with minor modifications [32]. A handheld colorimeter was used to measure the L*, a*, and b* values perpendicular to the surface of the crab cakes. Each set of samples was tested three times, and the average values were calculated. W (whiteness) was calculated using Equation (3).

(3) $$\mathrm{Whiteness}\left(W^{*}\right)=100-{\left[{\left(100-L^{*}\right)}^{2}+{a^{*}}^{2}+{b^{*}}^{2}\right]}^{\frac{1}{2}}$$

### 2.11. Electronic Nose Analysis

Following Yang’s method with minor modifications [33], the crab cake samples were crushed, and 2.50 g of the sample was accurately weighed into a 20 mL headspace vial. After sealing, the vial was left at room temperature for 30 min, and headspace analysis was performed using an electronic nose. Test conditions included the following: sensor cleaning time of 100 s, measurement time of 180 s, and injection flow rate of 300 mL/min. Three samples were selected per group and analyzed in triplicate; the results were averaged. Information on the electronic nose sensors is shown in Table 1.

### 2.12. Electronic Tongue Analysis

Following the method described by Cai et al., samples were prepared, and their odor characteristics were measured [34]. Thirty grams of crushed crab cake was placed in a beaker, and 150 mL of deionized water was added. The mixture was homogenized for 2 min, and then centrifuged at 7000× *g* for 20 min. In total, 35 mL of the supernatant was transferred to beakers designated for the electronic tongue, each labeled for testing. By simulating the taste perception mechanism of living organisms, the system evaluates the five basic tastes (sour, astringent, bitter, salty, and umami), with the taste thresholds for salty and sour set at −6 and −13, respectively, while the thresholds for all other indicators are set at 0. Each sample was analyzed in triplicate, and the data were analyzed using the instrument’s built-in database.

### 2.13. Sensory Evaluation

Sensory evaluations of the baked crab cakes were conducted using methods described in the relevant literature, with minor modifications [35,36]. A sensory panel consisting of 10 experienced sensory evaluators (with a 1:1 male-to-female ratio), all of whom had professional backgrounds in food science, was selected to perform the sensory analysis of the crab cakes. After baking, the crab cakes were cooled to room temperature, cut into small pieces, and placed on white plates for evaluation by the sensory panel. During formal evaluation, all samples were coded with three-digit random numbers, and the panelists were blinded to the treatment information. Each sample was evaluated in duplicate, with a 30-min interval between sessions, during which panelists rinsed their mouths with purified water to eliminate residual effects. Samples were presented in a randomized order to avoid order effects. The panel scored the crab cakes on color, odor, texture, and taste/mouthfeel according to Table 2, with a maximum score of 100 points.

### 2.14. Statistical Analysis

Continuous variables were expressed as the mean ± standard deviation (SD). Normality was verified by the Shapiro–Wilk test, and homogeneity of variances was confirmed by Levene’s test. One-way analysis of variance (ANOVA) followed by Duncan’s multiple range test was used for multiple-group comparisons, with different letters indicating significant differences among groups (*p* < 0.05).

## 3. Results and Discussion

### 3.1. Effect of HHP on the Potential Allergenicity of Crab Cakes

TM is a major allergen in crustacean seafood. Consequently, it can form specific IgE-mediated binding with the serum of 72% to 98% of individuals with crustacean food allergies, triggering severe allergic reactions [25]. However, due to significant individual variability in patient serum, difficulties obtaining serum samples due to limited patient availability and ethical constraints, and issues with component interference [18,25], in vitro sensitization assessments often use commercial monoclonal antibodies to enhance standardization and reproducibility. Therefore, this study used rabbit anti-TM polyclonal antibodies to determine the IgG binding capacity of crab cakes after treatment at 200 MPa for various durations.

**Figure 1:**
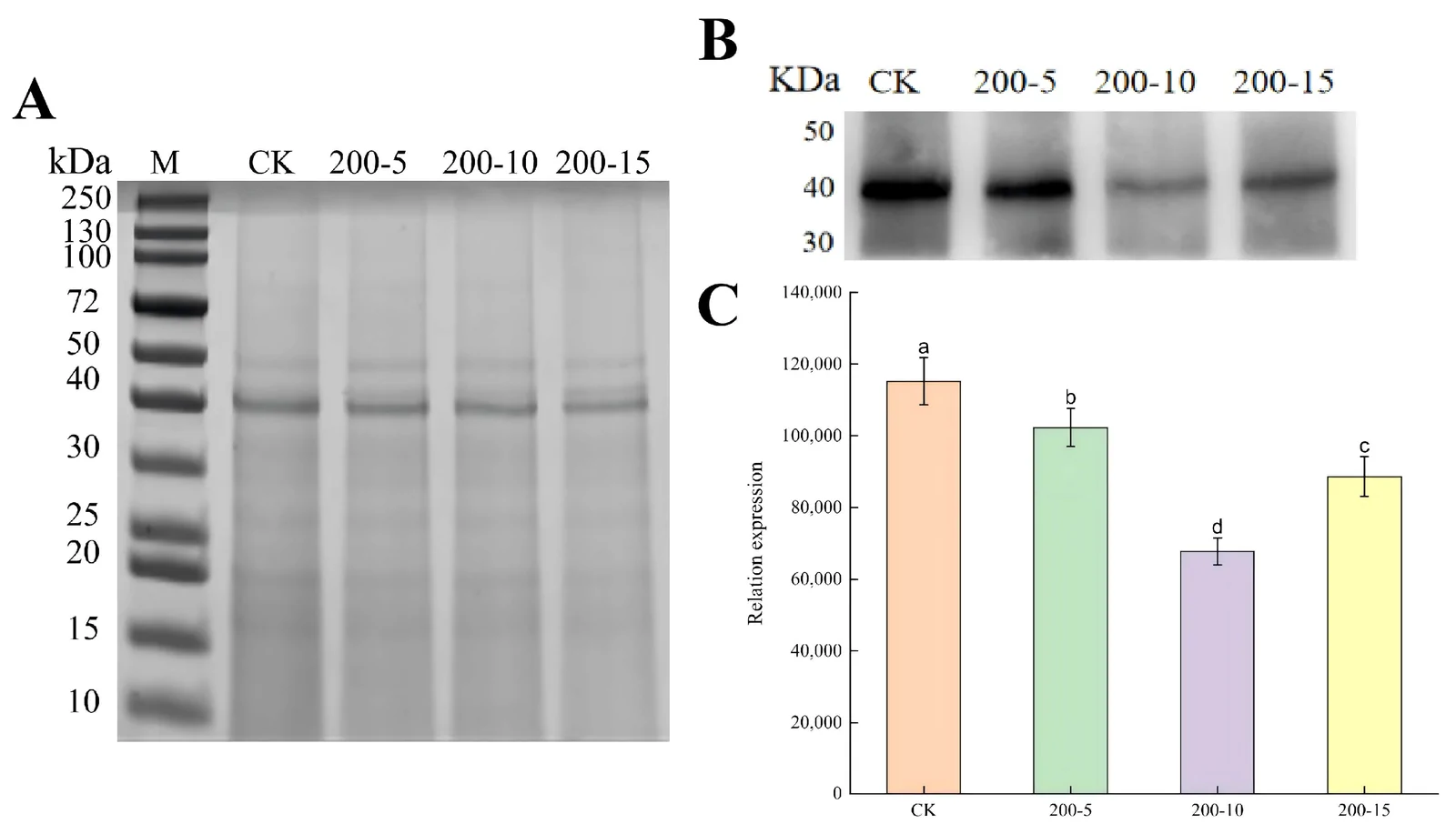
The effects of different treatment methods on the total protein loading (**A**) and the IgG-binding activity of TM (**B**,**C**) in crab cakes. Different lowercase letters represent significant differences (*p* < 0.05) between groups.

First, the effects of 200 MPa treatment for different durations (5, 10, and 15 min) on the protein components of crab cakes were analyzed via SDS-PAGE (Figure 1A). The results showed that, compared with the CK group, the total protein band profiles and grayscale intensities of the crab cake crude extracts were generally comparable across different HHP treatment times, suggesting that 200 MPa treatment had no significant effect on the major components or the overall content of proteins in the crude extract. On this basis, the effects of different treatment times on the IgG-binding capacity of TM were further evaluated (Figure 1B,C). Compared with the CK group, the grayscale values of samples treated at 200 MPa for different durations were all significantly reduced (*p* < 0.05), with the lowest grayscale value observed in the sample treated at 200 MPa for 10 min. This indicates that this treatment condition can significantly reduce the specific antibody recognition activity.

Combined with Figure 1A, under the premise of comparable total protein content, we speculate that this change may result from HHP-induced unfolding and refolding of protein secondary and tertiary structures, which destroy or mask the original surface conformational epitopes. These epitopes are the key sites for specific antibody recognition and binding, and the loss of their spatial integrity directly leads to a decrease in immunobinding capacity [22,37]. This is consistent with the findings of Yang et al., who reported that treatment at 600 MPa for 10 min significantly reduced the immunological activity of ovalbumin (OVA), suggesting that ultra-high pressure follows similar regulatory mechanisms in altering the spatial epitopes of food allergens [38]. Notably, when the treatment time was extended to 15 min, the grayscale values of the samples rebounded significantly compared with those at 10 min. Combined with the SDS-PAGE results, this might be attributed to the excessive aggregation or irregular refolding of denatured proteins under prolonged pressure, leading to the re-exposure of originally hidden linear epitopes or the formation of new potential antigenic epitopes [39]. Nevertheless, the immunological activity of the 200-15 group remained significantly lower than that of the CK group, demonstrating an inhibitory effect on the immunogenicity of TM. The above results indicated that the reduction in TM IgG immunoreactivity was most significant after treatment at 200 MPa for 10 min.

**Figure 2:**
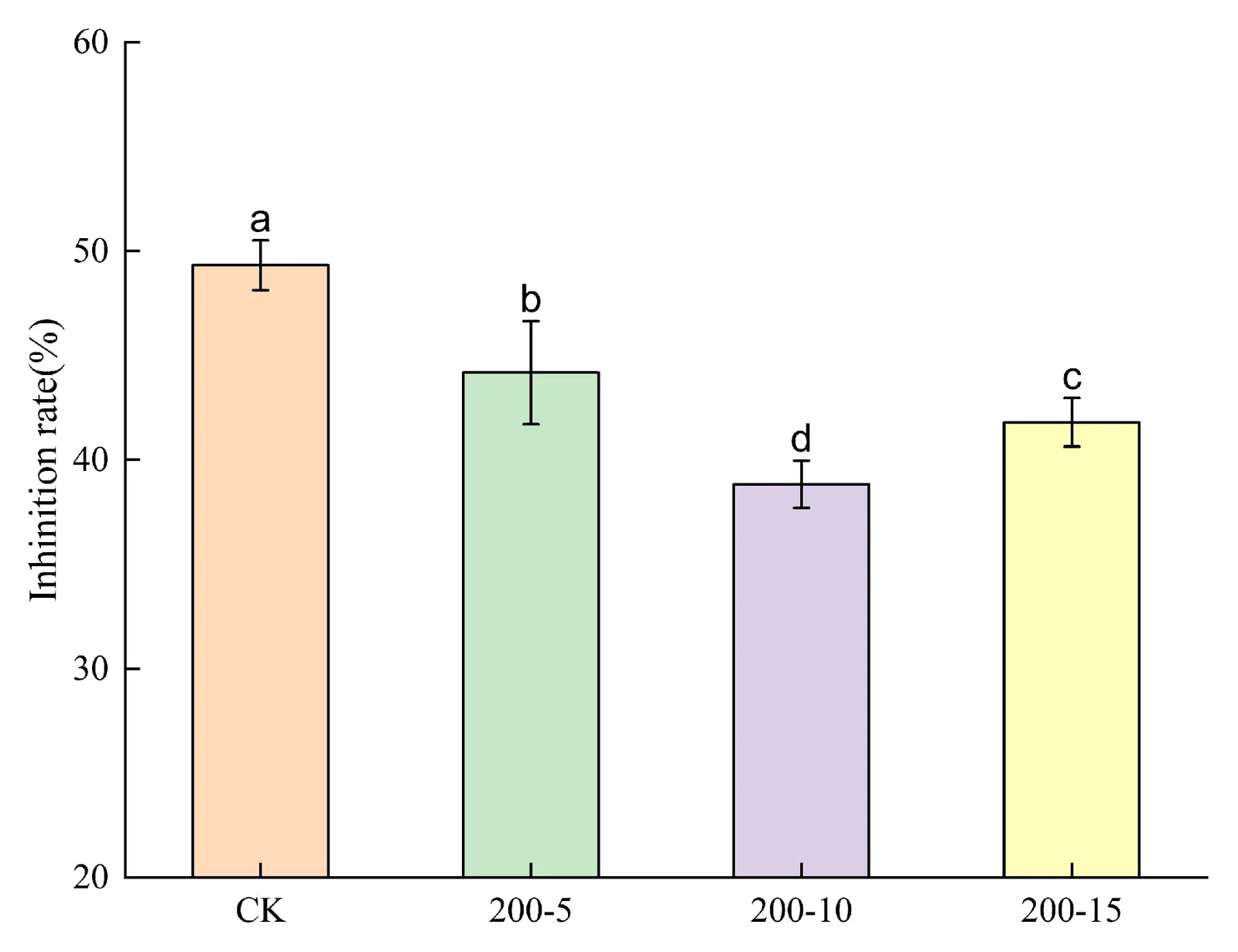
The effects of different treatment methods on the competition inhibition ELISA analysis of crab cakes. Different lowercase letters represent significant differences (*p* < 0.05) between groups.

An indirect competitive ELISA (ic ELISA) was used to evaluate further the effect of HHP on the IgG immunoreactivity of TM in crab cake. The results, shown in Figure 2, are consistent with the Western blot findings, indicating that regardless of whether conformational or linear epitopes were detected, the effect of HHP treatment on TM immunoreactivity followed the same time-dependent pattern, further supporting the reliability of the observed effects. Compared with the CK group, the competitive inhibition rate of TM by specific antibodies was significantly reduced by 21.19% following 10 min of treatment at 200 MPa. This indicates that HHP treatment significantly reduced the binding capacity of TM in crab cakes to specific antibodies. We speculate that this reduction in immunoreactivity may result from ultra-high pressure-induced unfolding or spatial rearrangement of TM protein conformation, which destroys or masks the natural antigenic epitopes [40]. This is consistent with the findings of Kang et al., who used a competitive inhibition ELISA to demonstrate that treating the peach allergen (Pru p 3 lipid transfer protein, LTP) at 500 MPa for 30 min led to a significant decrease in its competitive inhibition rate [41]. However, IgG reactivity rebounded significantly after 15 min, consistent with the Western blot (WB) results. This phenomenon suggests that some linear epitopes, originally masked by conformational changes, may have been re-exposed on the protein surface or may even have formed new antigenic epitopes, thereby leading to increased IgG immunoreactivity [40]. Nevertheless, the IgG-binding capacity in this treatment group remained significantly lower than that in the control group (CK) (*p* < 0.05). The results indicate that a 10-min treatment at 200 MPa can effectively reduce the binding capacity of crab cake TM to specific IgG.

### 3.2. Effect of HHP on the TM Immunoreactivity of Crab Cakes Following In Vitro Simulated Gastrointestinal Digestion

High resistance to digestion and prolonged retention in the gastrointestinal tract can increase the sensitizing potential of food allergens. Thus, the sensitizing potential of an allergen can be indirectly assessed based on its digestive stability in the gastrointestinal tract [42,43]. To determine the IgG-binding activity of HHP-treated crab cake TM using the ic Dot Blot method, this study established an in vitro gastrointestinal fluid-simulated digestion model. Since digested proteins exist as a mixture of peptide fragments, the indirect competitive Dot blot (ic Dot blot) method allows for immunological detection, making it suitable for screening the immunoreactivity of residual epitopes [44].

**Figure 3:**
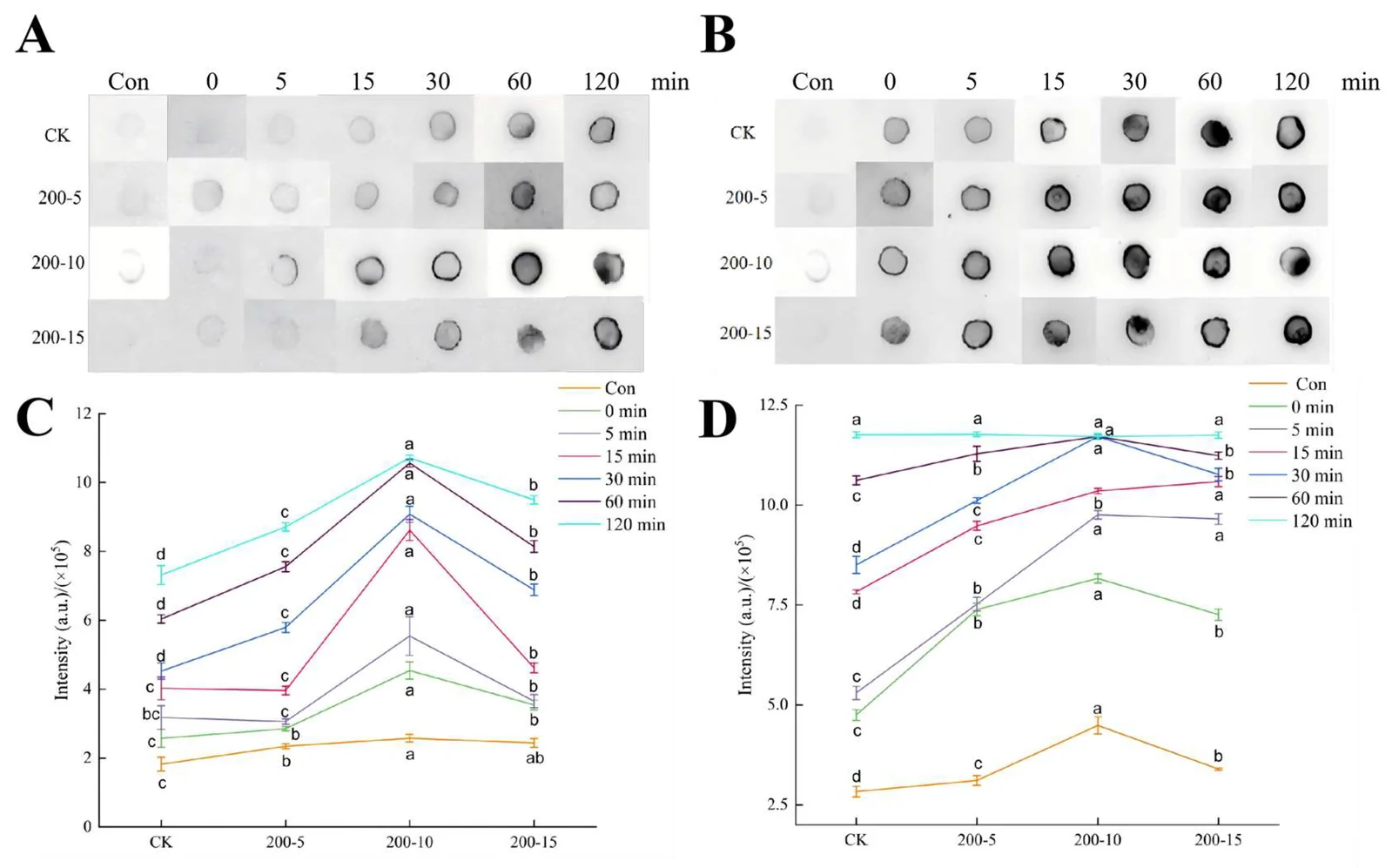
Immunoreactivity and relative expression levels of simulated gastrointestinal digestion of crab cakes subjected to different processing methods. Notes: (**A**): Dot blot analysis of gastric digests; (**B**): Dot blot analysis of intestinal digests; (**C**): relative expression levels of gastric digests; (**D**): relative expression levels of intestinal digests. Values of different groups with different lowercase letters (a–d) are significantly different at *p* < 0.05.

The results showing the effect of different HHP treatment durations on the gastrointestinal fluid digestibility stability of TM in crab cakes are presented in Figure 3A,C. At 0 min, compared with the CK group, the grayscale values in all HHP-treated groups were significantly higher (*p* < 0.05). This indicates that HHP treatment can significantly reduce the IgG-binding capacity of TM in the initial crab cakes [40,45]. As digestion time increased, the grayscale values of the spots in all groups rose significantly, indicating that the digestive stability of TM in all groups decreased with prolonged digestion. Specifically, during the 30–120-min digestion process, the grayscale values were ranked from highest to lowest as follows: the 200-10 group > the 200-15 group > the 200-5 group > the CK group. Significant differences were found between groups (*p* < 0.05). Notably, this indicated that the 200 MPa, 10-min treatment significantly reduced the digestive stability of TM [46]. This is consistent with the findings of Yang et al., whose study showed that HHP treatment promoted the enzymatic hydrolysis of ovalbumin (OVA) during in vitro digestion, indirectly suggesting that pressure treatment increases protein digestibility [38].

The digestive stability of TM in crab cakes is shown in Figure 3B,D. As the digestion time increased, the IgG-binding capacity of TM in all groups continued to decline. Ultimately, at 120 min of digestion, there were no statistically significant differences in gray values among the four sample groups (*p* > 0.05). This indicates that under prolonged trypsin action, the TM in all samples was eventually completely degraded into small peptide fragments, and the original conformation-dependent epitopes were almost entirely lost [47]. Notably, the 200-10 group exhibited the highest grayscale values at 30 and 60 min of digestion, indicating that its degradation rate was faster than that of the other groups (*p* < 0.05). However, after 120 min of digestion, the grayscale values stabilized across all groups, suggesting that by this time, the TM had likely been almost completely degraded into immunologically inactive peptide fragments. This is consistent with the findings of Liu et al., who reported that moderate ultra-high-pressure (300 MPa) treatment of crayfish TM effectively disrupted its α-helical structure, thereby increasing its susceptibility to trypsin [48].

Based on these results, we propose the following hypothesis: HHP treatment effectively induces partial unfolding of the coiled-coil dimeric structure of TM. While disrupting or masking key conformation-dependent IgG-binding epitopes, this process exposes more protease cleavage sites within the molecule, thereby enhancing its rate of digestive degradation in a simulated gastrointestinal fluid environment and significantly reducing the recognition capacity of crab meat TM by specific antibodies [47,49,50]. Among these treatments, the 200 MPa, 10-min HHP treatment was the most effective, as evidenced by the highest grayscale values during digestion, indicating the most substantial reduction in TM stability and the most rapid degradation under simulated gastrointestinal conditions.

### 3.3. Effect of HHP on the Water-Holding Capacity and Moisture Migration in Crab Cakes

**Figure 4:**
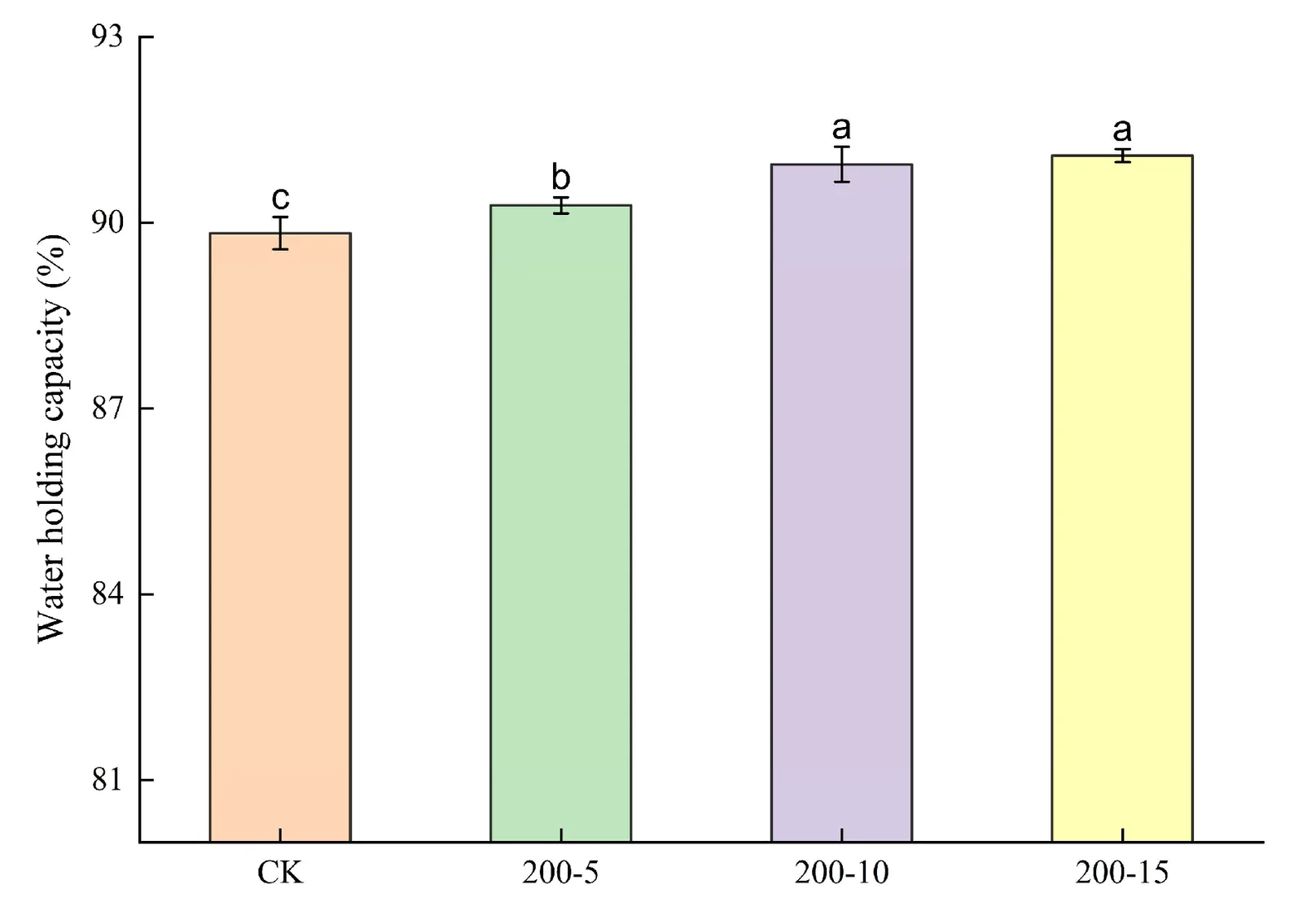
Water-holding capacity of crab cakes following different processing methods. The same letter represents no cant difference between groups (*p* > 0.05), and different letters represent sig differences between groups (*p* < 0.05).

Figure 4 shows the changes in WHC of crab cakes after HHP treatment. Compared with the CK group, the WHC of the crab cakes exhibited a significant upward trend (*p* < 0.05) as the treatment time at 200 MPa increased; the WHC of the 200-10 group reached its maximum value (90.94%). This is because HHP treatment alters the spatial structure of myofibrillar proteins in crab meat, exposing hydrophobic groups and enhancing their ability to bind water, and disrupts the structure of starch and dietary fiber (starch and dietary fiber derived from potato and carrot additives) [51,52,53]. HHP breaks the polymer chains of starch molecules, promoting the penetration of water molecules into the starch interior and the formation of new hydrogen bonds, thereby enhancing the water absorption and WHC of starch [54]. It also disrupts the surface structure of dietary fiber, exposing more hydrophilic groups and inducing a conversion to soluble dietary fiber (SDF). Notably, SDF contains more free hydrophilic groups, which can form a stronger binding network with water molecules, thereby improving overall WHC [54,55]. This is consistent with Chen et al., who reported that moderate ultra-high-pressure treatment induces cross-linking and gelation of myofibrillar proteins in cod mince, forming a three-dimensional network structure with greater WHC, resulting in a 27.40% increase in its water-holding capacity [56].

**Figure 5:**
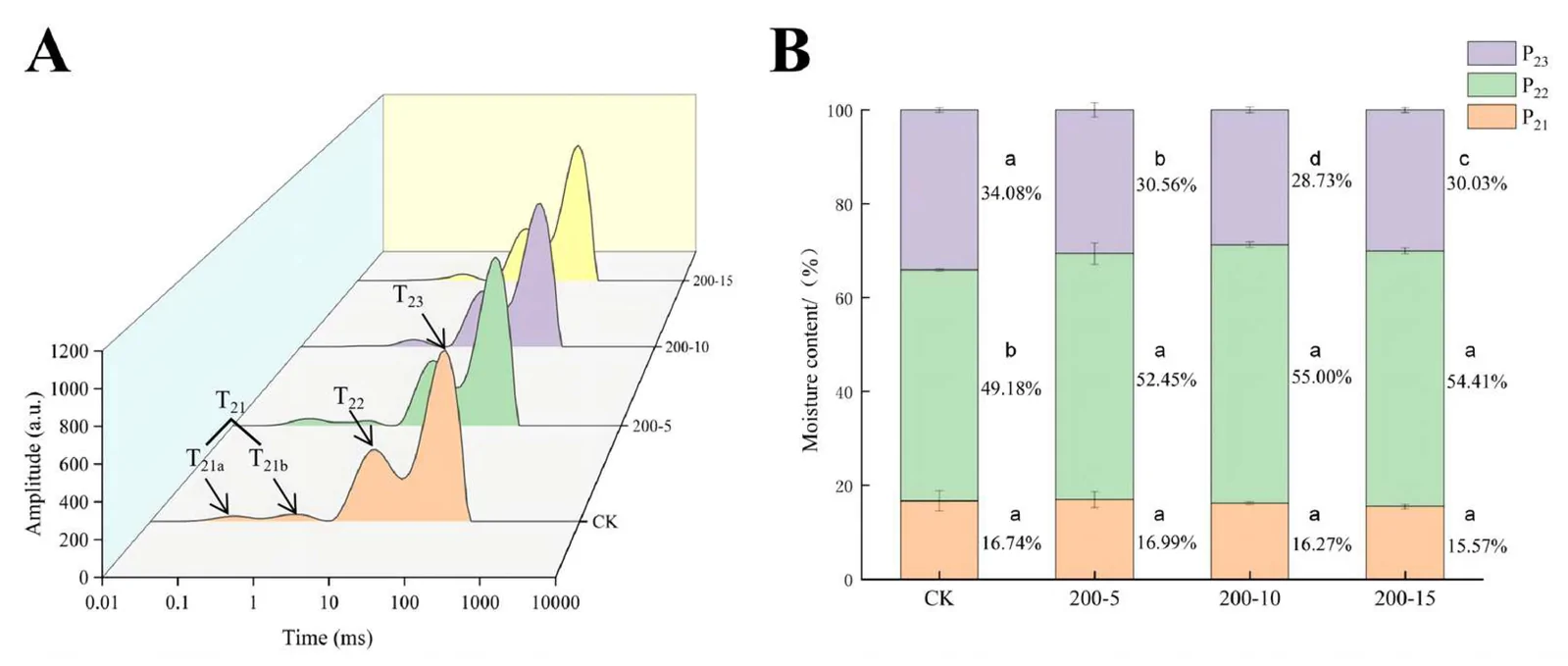
The effects of different treatment methods on the relaxation time (**A**) and water composition (**B**) of crab cakes. The same letter represents no cant difference between groups (*p* > 0.05), and different letters represent sig differences between groups (*p* < 0.05).

Low-field nuclear magnetic resonance (LF-NMR) analysis characterizes the distribution and migration patterns of water in a sample by detecting the relaxation behavior of hydrogen protons [57]. As shown in Figure 5A, the LF-NMR spectrum of the crab cake exhibits a total of four distinct proton peaks: strongly bound water with a relaxation time of 0.1–1 ms (T21a, water strongly bound to proteins); weakly bound water with a relaxation time of 1–10 ms (T21b, water weakly bound to proteins); water with limited mobility with a relaxation time of 1–100 ms (T22, water trapped within myofibrils); free water with relaxation times of 100–1000 ms (T23, water in the extracellular fluid) [58,59]. Among these, T21a and T21b together constitute protein-bound water (T21), with relaxation times ranging from 0.1 to 10 ms. Specifically, following HHP treatment, the T21a peaks in the 200-10 and 200-15 groups shifted to the right, while the T22 peaks shifted to the left. This indicates that after HHP treatment, strongly bound water in the crab cakes was converted to weakly bound water, the mobility of immobilized water was further restricted, and the overall fluidity of the water in the crab cakes decreased.

All four sets of samples exhibited proton peaks (T21, T22, T23) representative of the three types of water. This indicates that HHP treatment did not alter the composition of water within the crab cakes but only changed the distribution of the water content among the different types. The specific composition and content of moisture in the crab cakes are shown in Figure 5B, where P21, P22, and P23 represent the relative proportions of bound water, immobilized water, and free water, respectively. In the crab cakes, immobilized water is the primary form of moisture; its mobility lies between that of bound water and free water, and it is prone to dynamic transformation. A larger integral area corresponding to immobilized water indicates a greater amount of moisture retained within the fiber structure, which can effectively improve the product’s water-holding capacity [60]. The bound water content (P21) following HHP treatment showed no significant change (*p* > 0.05). In contrast, the content of water with limited mobility (P22) was significantly higher than that of the CK group (*p* < 0.05). This indicates that HHP treatment can convert free water into water with limited mobility, enhancing the interaction between water and the matrix and forming a denser, more uniform gel network [61]. The CK group had the highest free water content (P23), which is prone to loss during processing and storage, leading to weight loss and a dry, woody texture in the samples. Additionally, it may weaken the gel network structure, resulting in a softer mouthfeel. In contrast, the 200-10 group exhibited the lowest P23 value (*p* < 0.05), indicating that HHP treatment most effectively reduced the proportion of mobile water and promoted water immobilization within the gel matrix [62].

In summary, the 200-10 group exhibited the lowest P23 and the highest P22 content, consistent with the finding that this group had the best water-holding capacity. This indicates that HHP treatment helps improve the water-holding capacity of crab cakes and can reduce the potential risk of juice loss during subsequent processing.

### 3.4. Effect of HHP on the Texture Properties and Color of Crab Cakes

**Figure 6:**
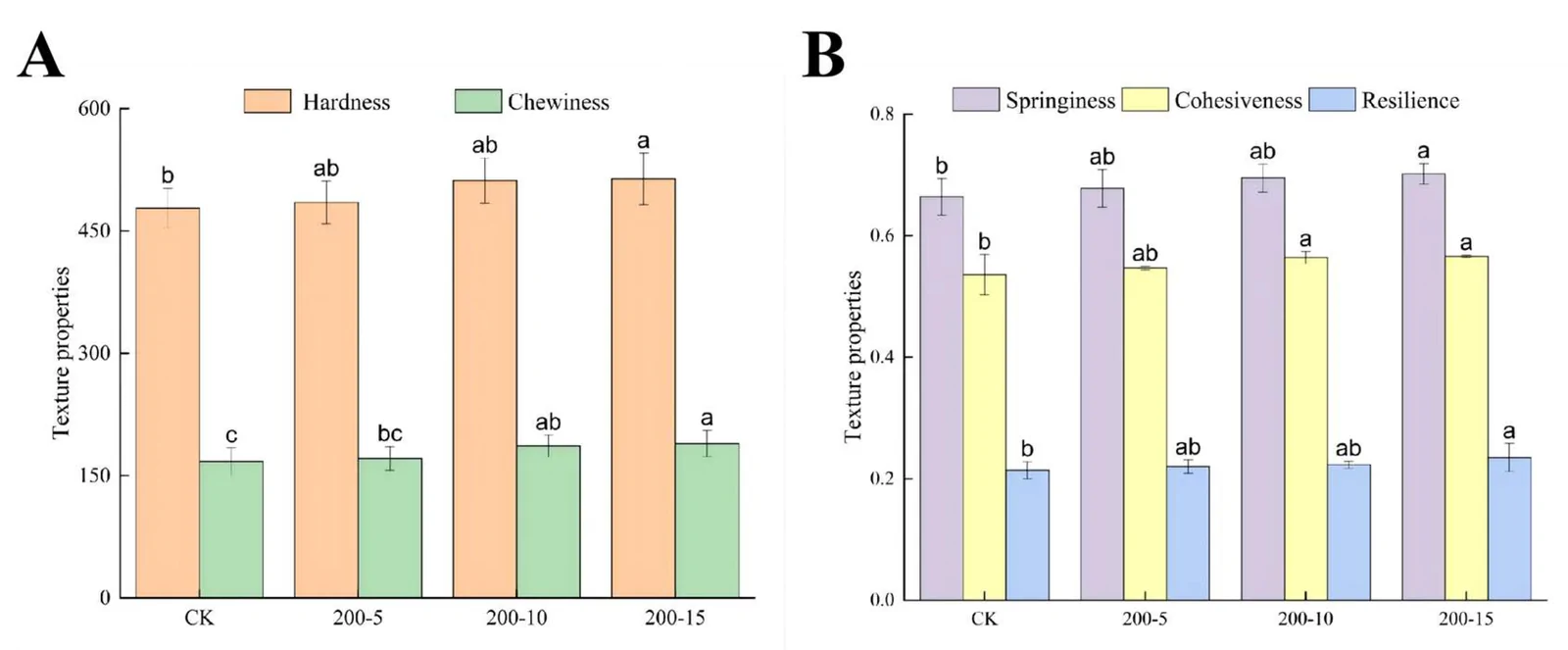
Effects of different processing methods on the texture characteristics of crab cakes. Notes: (**A**): Effects of different treatments on the hardness and chewiness of crab cakes; (**B**): Effects of different treatments on the springiness, cohesiveness, and resilience of crab cakes. The same letter represents no cant difference between groups (*p* > 0.05), and different letters represent sig differences between groups (*p* < 0.05).

Texture profile analysis is an important indicator of food quality and sensory acceptance [63]. The effects of different HHP treatments on the textural properties of crab cakes are shown in Figure 6. Hardness and chewiness are presented in Figure 6A, while springiness, cohesiveness, and resilience are shown in Figure 6B. Compared with the CK group, all textural parameters in the 200-15 group were significantly higher. Regarding hardness, this is attributed to the denaturation or aggregation of proteins under high pressure during HHP treatment, which affected the final gel network structure and enhanced the compactness and deformation resistance of the gel network [64,65]. Simultaneously, more glutamine and lysine residues, originally hidden within the molecules, were exposed, allowing the TG enzyme (derived from the excipient) to accelerate covalent cross-linking with proteins through an amide transfer reaction, thereby significantly enhancing the compactness of the gel network [66,67]. Furthermore, HHP disrupts starch crystals (derived from the potato puree), promotes the dissolution of amylose, and accelerates interactions between starch molecules, further enhancing hardness [68,69]. In terms of elasticity, HHP promotes moderate denaturation and rearrangement of myofibrillar proteins, which better maintained the gel matrix, and TGase-mediated covalent cross-linking contributed to the formation of a more elastic gel network [66]. Notably, in pairwise comparisons among the HHP-treated groups, no significant differences were observed in any textural parameters except chewiness (*p* > 0.05). The chewiness of the 200-10 and 200-15 groups was significantly higher than that of the CK group. This is because chewiness is a comprehensive sensory parameter determined jointly by hardness, cohesiveness, and elasticity [70]; the 200-15 group exhibited significantly higher values in all three individual parameters compared with the CK group, thus resulting in significantly higher chewiness (*p* < 0.05). In contrast, although the 200-10 group showed no significant differences from the CK group in these three individual textural parameters, the combined effect of the three parameters resulted in a significant difference in chewiness (*p* < 0.05). In summary, the 200 MPa treatment for 15 min contributed to improving the gel structural integrity of the crab cakes, while the 10 min treatment also effectively enhanced the textural properties.

**Figure 7:**
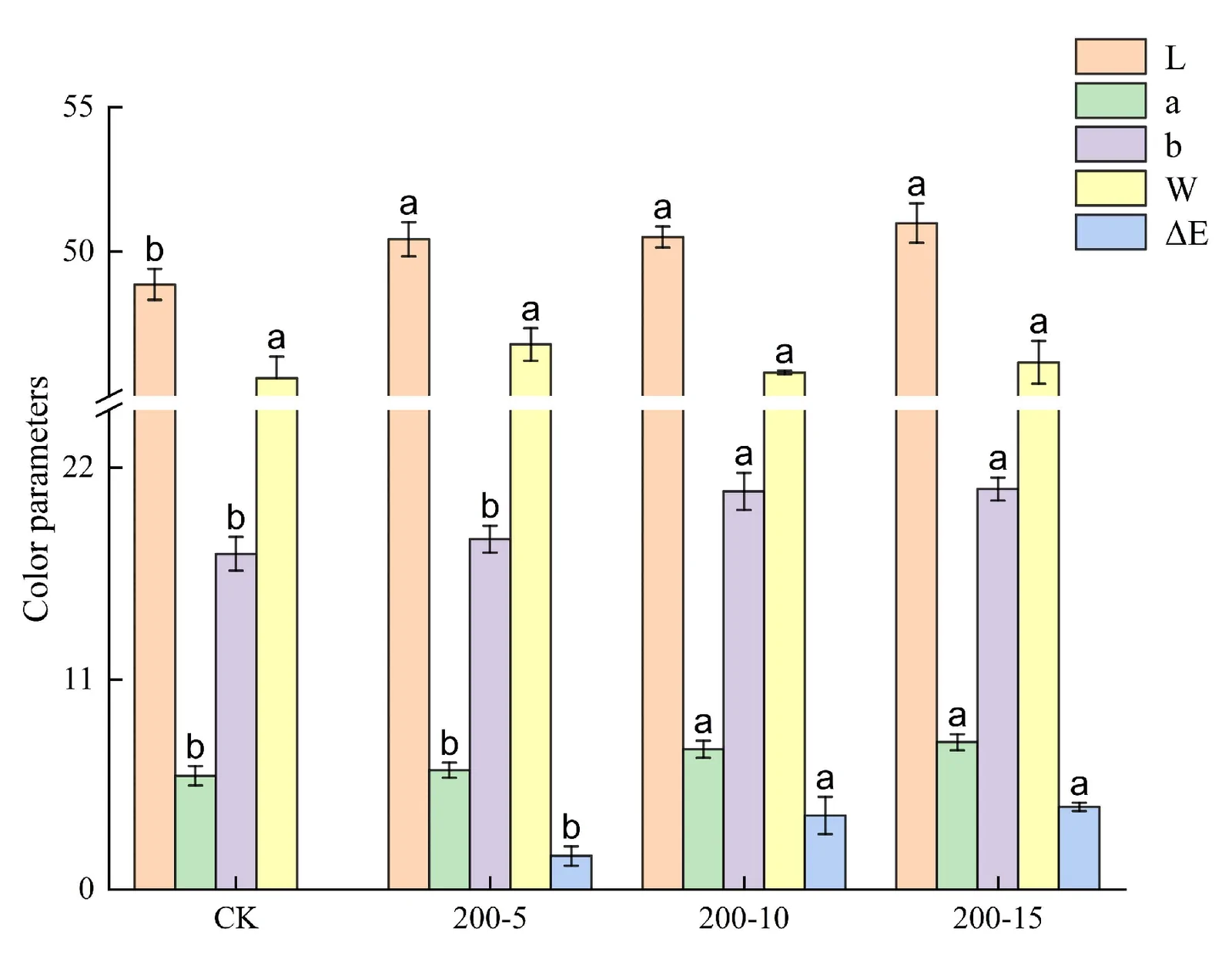
Effects of different processing methods on the color difference in crab cakes. The same letter represents no cant difference between groups (*p* > 0.05), and different letters represent sig differences between groups (*p* < 0.05).

Color is one of the key indicators for evaluating food quality and directly influences sensory acceptance and a product’s market value [71]. As shown in Figure 7, the L* (lightness), a* (redness), and b* (yellowness) values of the 200-10 and 200-15 groups were significantly higher than those of the CK group. This may be due to HHP inducing conformational changes in proteins, leading to the exposure of chromophores or molecular rearrangement, thereby altering light absorption and reflection [72,73]. Furthermore, under high pressure, the cell walls of plant tissues in the fillers (potatoes and mashed carrots purees) ruptured, allowing endogenous pigments to be released more fully into the protein matrix. This increased the samples’ a* and b* values, giving the crab cakes a deeper reddish-yellow hue overall [74]. On the other hand, the increase in the a* value may also result from high pressure promoting the conformational rearrangement of astaxanthin–protein complexes, releasing free astaxanthin and thereby enhancing redness [75]. In contrast, the significant rise in the b* value may be associated with pressure-induced denaturation and oxidation of hemocyanin [76]. This differs from the findings of Enchangan et al., whose study showed that treating Indian mackerel at 400 MPa for 20 min affected the structural integrity of the proteins, resulting in a significant decrease in the sample’s b* value (*p* < 0.05) [77]. Notably, this discrepancy may stem from differences in the study systems: their system consisted of pure myofibrillar protein without the introduction of external pigments; simultaneously, HHP promoted uniform protein aggregation, increasing light reflectance (W), which led to a decrease in the b* value. In contrast, the crab cakes in this study constitute a complex, multicomponent system, and under the influence of HHP, these components collectively contributed to an increase in the b* value. It is worth noting that there was no significant difference in the W value of the crab cakes before and after treatment (*p* > 0.05), consistent with the conclusion reported by Enchangan et al. that HHP treatment at 150–250 MPa does not significantly alter the whiteness of crab meat [77]. Although the L*, a*, and b* values changed significantly before and after treatment, since W represents the combined effect of the opposing changes in these parameters, the changes offset each other, resulting in no significant difference in the W value. In contrast, the total color difference (ΔE), as a cumulative index, showed a significant difference and captured the overall color shift more sensitively. In summary, HHP treatment resulted in a richer overall color in the crab cakes.

### 3.5. Effect of HHP on the Electronic Nose for Crab Cakes

E-nose data primarily reflect the composition and relative concentrations of volatile flavor compounds in food and serve as an important tool for evaluating flavor quality [78]. All treatment groups exhibited high responses to broad-spectrum aldehydes/ketones (Sensor2), nitrogen-containing compounds (Sensor10), and organic compounds (Sensor12), likely related to the formation of complex heat-processed aromas resulting from the Maillard reaction during the crab cake production process [79]. In terms of overall response values, no marked differences were observed between the HHP-treated groups and the CK group based on the sensor signal profiles, suggesting that HHP treatment largely preserved the original flavor characteristics of the crab cakes.

**Figure 8:**
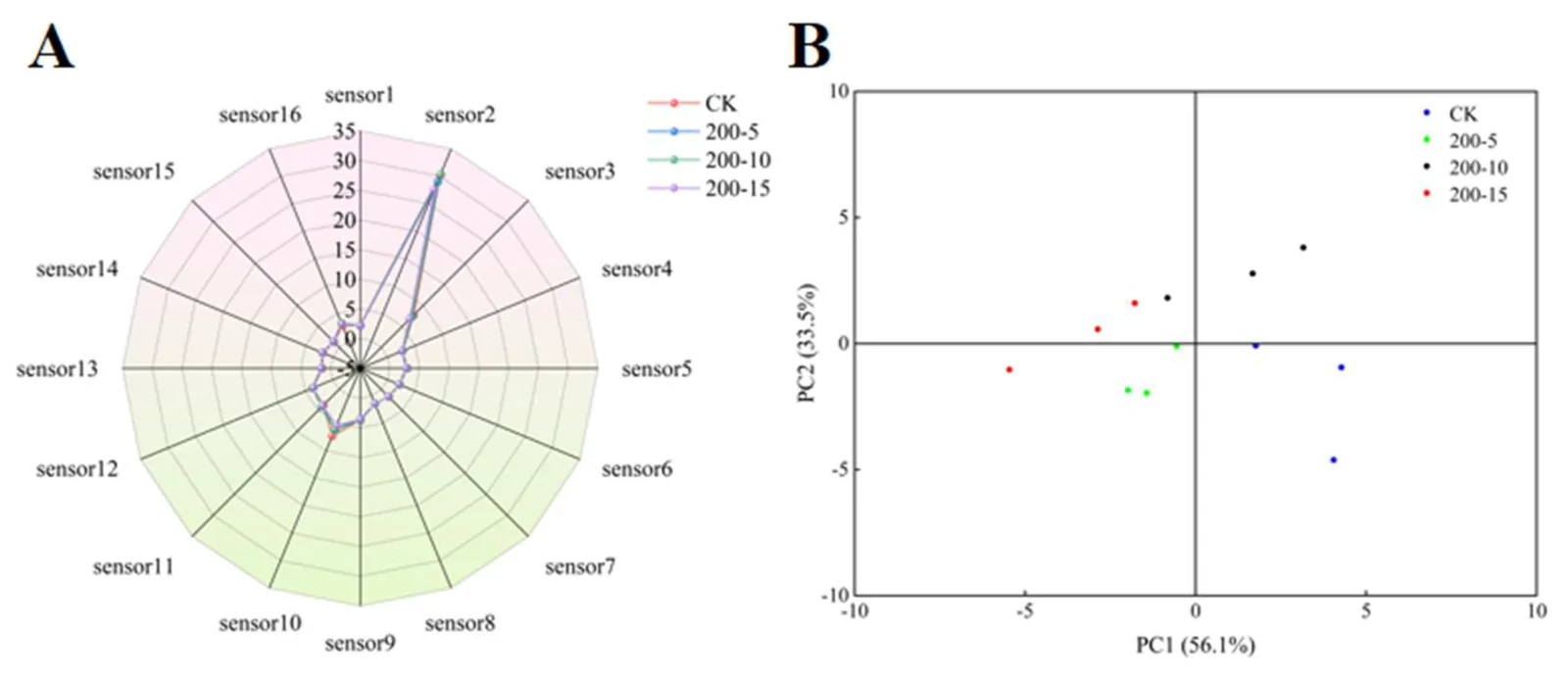
The effects of different treatment methods on the electronic nose radar chart (**A**) and principal component analysis (**B**) of crab cakes.

Principal component analysis (PCA) was performed as an exploratory multivariate visualization method to provide an intuitive overview of the E-nose data in Figure 8B. PC1 and PC2 contributed 54.9% and 34.0% of the variance, respectively, with a cumulative variance contribution of 88.9%, effectively reflecting the core information of the volatile compounds in the crab cakes under HHP treatment. Among these, PC1 played a dominant role, and the overlapping regions of the sample groups in the two-dimensional space reflected the overall similarity and differences in odor [80]. Specifically, the 200-10 group showed a large degree of overlap with the control group (CK group) on the principal component 1 (PC1) axis, indicating that the 200-10 group’s composition of primary volatile flavor compounds was closest to that of the CK group. Meanwhile, the 200-5 group showed partial overlap with the CK group along the PC2 axis, suggesting a high degree of similarity in the composition of secondary flavor components. Overall, the HHP-treated groups and the CK group exhibited varying degrees of overlap, indicating that HHP treatment had a minimal disruptive or modifying effect on the overall odor profile of the crab cakes. Furthermore, it largely preserved their inherent flavor characteristics and original aromatic quality.

### 3.6. Effect of HHP on the Electronic Tongue for Crab Cakes

**Figure 9:**
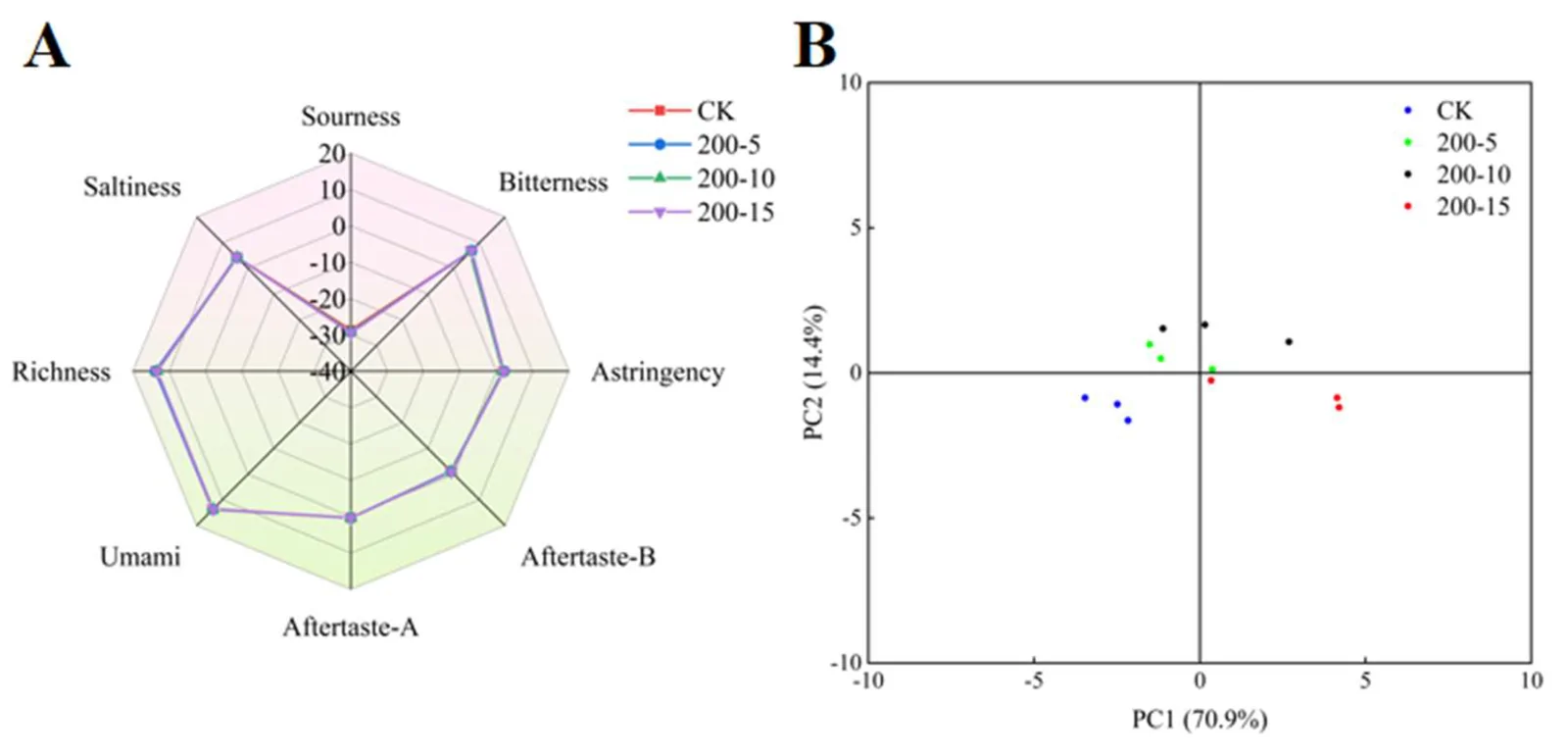
The effects of different treatment methods on the E-tongue radar chart (**A**) and principal component analysis (**B**) of crab cakes.

Protein denaturation and changes in tissue structure caused by HHP may directly affect the release of flavor compounds. Therefore, we further used an electronic tongue to quantitatively characterize the overall flavor profile of the crab cakes [48]. As shown in the flavor radar plot from the electronic tongue in Figure 9A, the sourness response values for all samples were below their taste threshold (−13), indicating that none of the samples exhibited sourness. Compared with the CK group, the flavor profiles of the various HHP-treated groups were similar. Since the physical effects of HHP do not typically break covalent bonds, small-molecule flavor compounds—such as free amino acids and flavor nucleotides—are largely preserved, contributing to the maintenance of the crab cakes’ inherent flavor. As shown in Figure 9B, PC1 (70.9%) and PC2 (14.4%) account for 85.1% of the cumulative variance, effectively reflecting the differences in flavor among the various crab cakes under HHP treatment. Among these, PC1 contributes significantly more than PC2 and plays a dominant role in distinguishing the samples [81]. Specifically, the 200-5 and 200-10 groups were closest to the CK group along the PC1 axis, indicating that these two treatment groups were most similar to the CK group in terms of the primary flavor characteristics that contributed most to the differences. In contrast, the 200-15 group was closer to the CK group along the PC2 axis, suggesting that its secondary flavor components were similar to those of the CK group. Overall, all HHP-treated crab cakes overlapped with the CK group to varying degrees, indicating that HHP had a minimal impact on the original flavor profile of the crab cakes.

### 3.7. Effect of HHP on the Sensory Evaluation of Crab Cakes

**Figure 10:**
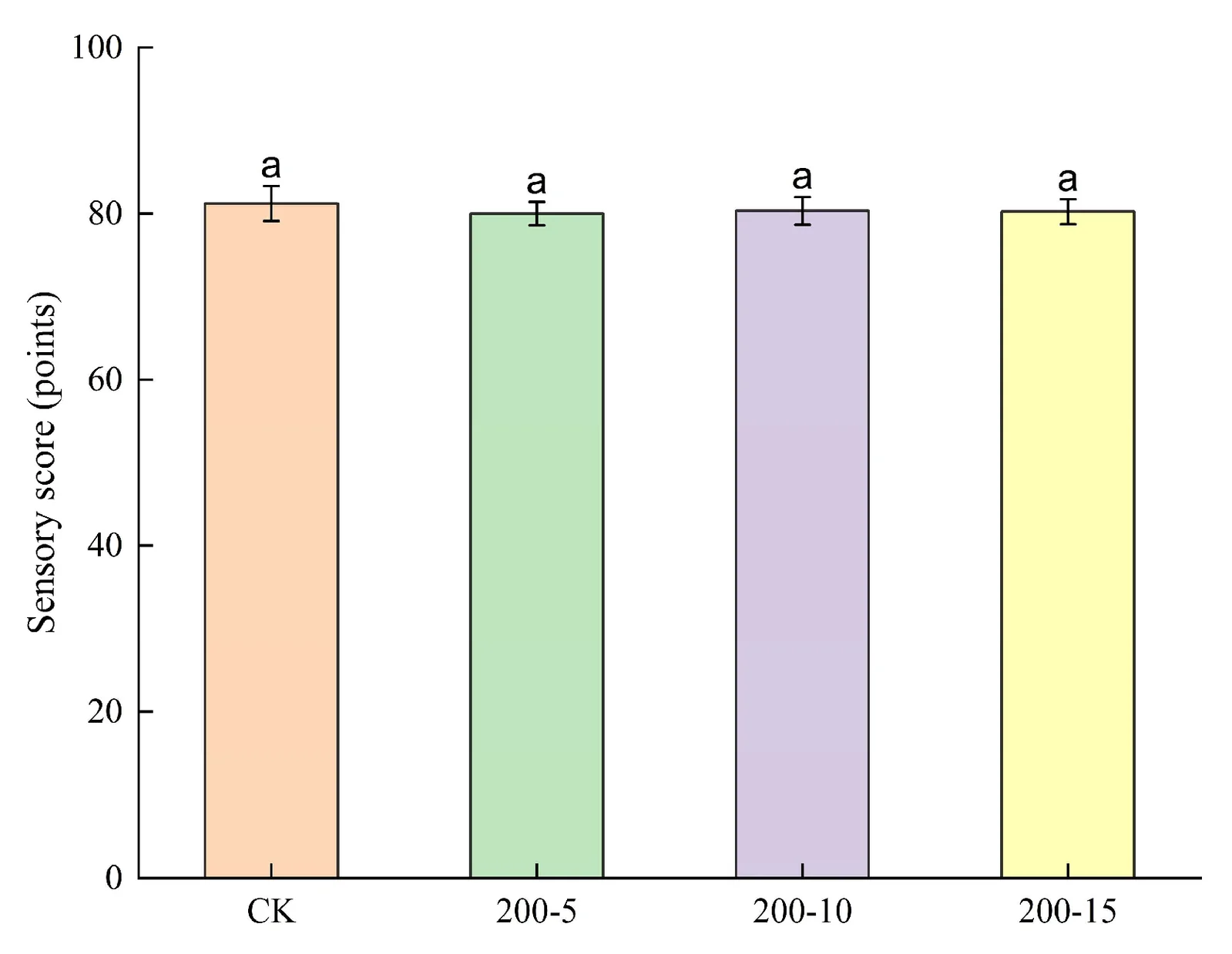
The effects of different treatment methods on the sensory evaluation of crab cakes. The same letter represents no cant difference between two treatment groups (*p* > 0.05).

Sensory evaluations were conducted on HHP-treated crab cakes to assess sensory acceptability by the study panel. (Figure 10). Compared with the CK group, there were no significant differences in the HHP-treated crab cakes (*p* > 0.05). This finding corroborates the results from the electronic nose and electronic tongue tests mentioned above, indicating that HHP treatment does not reduce the food’s original sensory acceptability. This is also consistent with findings from related studies on seafood products such as shrimp paste and pomfret fillets, which showed that their overall sensory acceptability remained close to that of untreated control groups after being subjected to 200 MPa treatment for varying durations [82,83].

## 4. Conclusions

Under the conditions tested in this study, the HHP treatment at 200 MPa for 10 min was identified as the most effective among the evaluated conditions for balancing the reduction in IgG-binding capacity and the overall quality of *Portunus trituberculatus* crab cakes. However, further optimization—including screening of additional pressure levels, treatment durations, independent production batches, and validation of microbiological safety—is warranted to establish a comprehensively optimized HHP process. Combined Western blot, ELISA, and Dot blot analyses revealed that this treatment effectively reduced the IgG-binding capacity of TM—the major allergen in crab cakes—by weakening its antibody recognition and accelerating its digestion in a simulated gastrointestinal environment. We speculate that the underlying mechanism may involve pressure-induced conformational changes, although this hypothesis requires further validation through structural biology studies.

At the same time, this process significantly improved the overall quality of the crab cakes. In terms of water-holding capacity, HHP altered the spatial structure of myofibrillar proteins and excipients (starch and dietary fiber), promoting the conversion of free water to bound water and significantly enhancing the water-holding capacity of the crab cakes. In terms of texture and color, HHP exposes active sites on proteins, promoting cross-linking by TGase, while simultaneously disrupting the crystalline structure of starch and enhancing the density of the gel network. It also significantly improves the color parameters of the crab cakes by inducing conformational changes in proteins and releasing endogenous pigments from the excipients. In terms of flavor and sensory quality, HHP treatment effectively preserves the inherent flavor and taste profile of the crab cakes, demonstrating good sensory acceptability while optimizing their physicochemical properties.

It is worth noting that the observed effects in this study are not solely attributable to HHP itself, but rather to the synergistic interaction between HHP and the complex food matrix (containing crab meat, vegetable puree, modified starch, and transglutaminase, among others). This feature represents a key distinction from studies using purified protein systems and enhances the practical relevance of the findings. In summary, this study provides experimental evidence and a theoretical basis for the application of HHP in reducing the immunoreactivity of crab products. However, further validation using crab-allergic patient sera, basophil activation tests, or appropriate animal models is still required to assess changes in IgE-mediated allergenicity, thereby enabling a more comprehensive evaluation of the clinical efficacy of HHP treatment.

## Figures and Tables

**Table 1:** E-Nose sensor names and performance descriptions.

No.	Sensor Name	Performance Description
**1**	Sensor1	Volatile hydrides
**2**	Sensor2	Broad-spectrum aldehydes and ketones
**3**	Sensor3	Broad-spectrum sulfides; inorganic and organic sulfur compounds
**4**	Sensor4	Methyl compounds; aromatic alkanes
**5**	Sensor5	Polycyclic aromatic hydrocarbons
**6**	Sensor6	Broad-spectrum alcohols, ethers, and acids
**7**	Sensor7	Broad-spectrum esters; nitrogenous compounds
**8**	Sensor8	Short-chain alkanes and alkenes
**9**	Sensor9	Aromatic ammonia compounds
**10**	Sensor10	Nitrogenous compounds
**11**	Sensor11	Broad-spectrum organic compounds
**12**	Sensor12	Methyl aromatics
**13**	Sensor13	Volatile sulfur compounds
**14**	Sensor14	Aromatic macromolecular compounds
**15**	Sensor15	Oxygen-containing organic compounds
**16**	Sensor16	Long-chain alkanes and alkenes

**Table 2:** Sensory scoring criteria.

Evaluation Item	Evaluation Criteria	Score
Color (20)	Golden yellow surface, uniform color	15–20
	Light yellow surface, slightly uneven color	8–14
	Dull surface, uneven color	1–7
Odor (20)	Rich crab aroma, harmonious flavor	15–20
	Moderate crab aroma, relatively less harmonious flavor	8–14
	No significant crab aroma, obvious off-odor	1–7
Texture (30)	Intact shape, not easy to disintegrate, no cracks	25–30
	Relatively intact shape, easy to disintegrate, slight cracks	13–24
	Fragmented shape, disintegrated, severe cracks	1–12
Taste and Mouthfeel (30)	Delicious, moderate saltiness, and good texture (neither too soft nor too hard).	25–30
	Relatively delicious, moderate saltiness, and acceptable texture.	13–24
	Poor taste, over/under-seasoned, and undesirable texture (too soft or too hard).	1–12

## Data Availability

The original contributions presented in the study are included in the article. Further inquiries can be directed to the corresponding author.

## Abbreviations

The following abbreviations are used in this manuscript:

HHP	High hydrostatic pressure
TM	Tropomyosin
ic ELISA	Indirect competitive ELISA
HDP	Hydroxypropyl di-starch phosphate
TGase	Transglutaminase
WHC	Water-holding capacity
SDF	Soluble dietary fiber
